# Retraction: C-reactive protein in aortic valve disease

**DOI:** 10.1186/1476-7120-4-42

**Published:** 2006-11-08

**Authors:** Pedro L Sanchez, Anna Maria Mazzone

**Affiliations:** 1 Associated Professor of Cardiology, Instituto de Ciencias del Corazón (ICICOR), Hospital Clínico Universitario de Valladolid, C/Ramón y Cajal n° 3, 47005, Valladolid, Spain; 2 Department of Cardiology and Cardiac Surgery, CNR Institute of Clinical Physiology, Ospedale Pasquinucci, Massa, Italy

A preliminary version of a review article [1] was published in error on 16 October 2006.

The correct version of the article [2] had previously been published in *Cardiovascular Ultrasound*.

The authors have agreed that the preliminary version [1] should be retracted.
